# The STS case study: an analysis method for longitudinal qualitative research for implementation science

**DOI:** 10.1186/s12874-021-01215-y

**Published:** 2021-02-05

**Authors:** Jennifer M. Van Tiem, Heather Schacht Reisinger, Julia E. Friberg, Jaime R. Wilson, Lynn Fitzwater, Ralph J. Panos, Jane Moeckli

**Affiliations:** 1 VA Office of Rural Health (ORH), Veterans Rural Health Resource Center-Iowa City, Iowa City VA Healthcare System, Iowa City, IA USA; 2 VA Health Services Research & Development Service, Center for Access and Delivery Research and Evaluation, Iowa City VA Health Care System (152), 601 Highway 6 West, Iowa City, IA 52246 USA; 3 The Department of Internal Medicine, University of Iowa Carver College of Medicine, Iowa City, IA USA; 4 Institute for Clinical and Translational Science, University of Iowa, Iowa City, IA USA; 5 VISN 10/Cincinnati Tele-CC System, Cincinnati, OH USA

**Keywords:** Longitudinal qualitative research, Science and technology studies, Implementation, Telemedicine, Critical care, Ethnography

## Abstract

**Background:**

Ethnographic approaches offer a method and a way of thinking about implementation. This manuscript applies a specific case study method to describe the impact of the longitudinal interplay between implementation stakeholders. Growing out of science and technology studies (STS) and drawing on the latent archaeological sensibilities implied by ethnographic methods, the STS case-study is a tool for implementors to use when a piece of material culture is an essential component of an innovation.

**Methods:**

We conducted an ethnographic process evaluation of the clinical implementation of tele-critical care (Tele-CC) services in the Department of Veterans Affairs. We collected fieldnotes and conducted participant observation at virtual and in-person education and planning events (*n* = 101 h). At Go-Live and 6-months post-implementation, we conducted site visits to the Tele-CC hub and 3 partnered ICUs. We led semi-structured interviews with ICU staff at Go-Live (43 interviews with 65 participants) and with ICU and Tele-CC staff 6-months post-implementation (44 interviews with 67 participants). We used verification strategies, including methodological coherence, appropriate sampling, collecting and analyzing data concurrently, and thinking theoretically, to ensure the reliability and validity of our data collection and analysis process.

**Results:**

The STS case-study helped us realize that we must think differently about how a Tele-CC clinician could be noticed moving from communal to intimate space. To understand how perceptions of surveillance impacted staff acceptance, we mapped the materials through which surveillance came to matter in the stories staff told about cameras, buttons, chimes, motors, curtains, and doorbells.

**Conclusions:**

STS case-studies contribute to the literature on longitudinal qualitive research (LQR) in implementation science, including pen portraits and periodic reflections. Anchored by the material, the heterogeneity of an STS case-study generates questions and encourages exploring differences. Begun early enough, the STS case-study method, like periodic reflections, can serve to iteratively inform data collection for researchers and implementors. The next step is to determine systematically how material culture can reveal implementation barriers and direct attention to potential solutions that address tacit, deeply rooted challenges to innovations in practice and technology.

**Supplementary Information:**

The online version contains supplementary material available at 10.1186/s12874-021-01215-y.

## Background

Ethnographic approaches offer both a method and a way of thinking about implementation science. As method, ethnography offers specific ways to document and track the implementation process in health services research. These include rapid cycle assessment [1, 2], periodic reflections [3], and pen portraits [4], which are based upon the triangulation of multiple, diverse data sources (i.e., participant observation, in-depth interviews, document review) [5, 6]. As a way of thinking, ethnography orients researchers and implementors to “everyday” contexts, which includes the local and the lived experience, as well as the tacit and implied [7, 8]. Applied to process evaluations [9–11], adaptation and tailoring [3], and facilitation [5], the primary contribution of an ethnographic approach to implementation science [12] is its comparative and holistic examination of people’s social worlds in relationship to newly introduced interventions.

We seek to contribute to the literature on ethnography in implementation science by illustrating an approach of the case study method that we believe is well-suited to describe the impact of the longitudinal interplay between implementation stakeholders. Case studies are a familiar way to present ethnographic findings related to implementation processes [13, 14]. In this article, we demonstrate a form of the case study method that grows out of science and technology studies (STS) and draws out the latent archaeological sensibilities implied by ethnographic methods [15–18]. Archeological insights are gleaned from attention to material culture, or the “stuff” with which people carry out the work of their everyday lives. Stories about how people carry out their lives with their stuff has been the work of ethnography since its inception as a method [19], but STS shifts the point of view of the narrator. Rather than stories told from the perspective of the human actors, STS starts with the material object and builds stories about the world based on how things and people share and shape each other through social practices [15, 20].

This kind of storytelling is familiar to doctors and nurses, who “expect the patient to tell a story about daily life-events in which entities of all kinds (beans, blood, table companions, cars, needs, sugar) coexist and interfere with one another” [16]. Writing an STS case study challenges researchers to “tell stories about medicine” that read like “a good case history” [16]. To illustrate the potential of this method, in this article we “recover archaeologically and interrogate ethnographically” part of the process of implementing critical care telemedicine (Tele-CC) in the Department of Veterans Affairs (VA) [21]. By tracing the Tele-CC implementation process through people’s use and manipulation of elements of material culture, we will ground our interpretation of our observations and interviews in some of the actual objects people handled every day in their interactions with Tele-CC. We engaged with sites through repeated brief encounters over several years. As a result, we will be able to describe the contextual shaping of Tele-CC implementation through time, as well as across sites at specific points in time.

We argue that this form of case study (termed an “STS case study”) is a novel form of longitudinal qualitative research (LQR) that allows implementors to understand and impact the implementation process by distilling a lot of diverse data [22, 23] into summaries and categories that make it possible to follow and understand change over time [23]. LQR is both a method for data collection and data analysis. Data collection based on LQR involves ethnographic engagement [24] and data analysis techniques requiring both cross-sectional and longitudinal examinations [22, 25]. Taken together, these data collection and analysis strategies make complexity digestible. Qualitative researchers in implementation science have picked up and used LQR to track adaptations through periodic reflections [3] and pen portraits [4]. Periodic reflections are a format for guided discussions, conducted over time, that serve as a record of an implementation effort [3]. A pen portrait organizes data from different sources, at different time points, together in one document; it is like a collage describing one site where an innovation is being implemented [4]. Both periodic reflections [26–29] and pen portraits [30, 31] have been used in the field to help develop study protocols; pen portraits have also been used as a method of data analysis [32, 33]. As a novel form of LQR, the STS case study method introduces the opportunity to engage with material culture, and thus contributes a way to focus and re-focus, or calibrate, the analytic lens, or to look for how local use and understanding of the material elements of an intervention changes over time, and what that could mean for the normalization [34–36] of the implementation as a whole. The aims of this paper are twofold: 1) to contribute to the literature on the role of ethnography in implementation science; and to achieve that by providing a case study about 2) tracing how Tele-CC and ICU staff negotiate the implementation of surveillance technology.

The goal of the VA Tele-CC program is to expand and improve the quality of critical care delivery. In 2011–2012, two Tele-CC programs launched in VA utilizing Philips eCareManager. Currently, two hubs with attendant satellite-hubs, serve approximately 30% of VA ICUs. In 2016, one of the two Tele-CC hubs in VA partnered with eight ICUs that were primarily lower-resourced, smaller, and located in geographically isolated rural hospitals that have been especially affected by the national shortage of critical care-certified physicians and nurses [37–39]. The VA Office of Rural Health (ORH) funded the provision of Tele-CC in these ICUs. Tele-CC includes bedside physiologic monitor upgrades, continuous monitoring, night and weekend tele-intensivist support, and on-demand support for emergency departments. It is a technological innovation that requires both the unidirectional flow of data inputs (e.g., vital signs and labs) from the bedside to the Tele-CC, as well as teamwork between ICU and Tele-CC staff to make decisions based on these inputs and provide care. Proprietary Philips algorithms built into the Tele-CC system alert Tele-CC staff to acute physiologic concerns (e.g., sepsis alert), and the Tele-CC staff then investigate by reviewing the inputs and connecting with the ICU staff.

Prior research has shown mixed results related to staff acceptance of Tele-CC [40]. Knowing this, external facilitators [41–43] built a community of practice around Tele-CC through commitment work [35, 44] characterized by a series of implementation strategies related to planning and education (i.e., building buy-in, developing relationships, developing materials, and educating) [45] that unfolded over time through virtual and in-person events. There were separate and coinciding technical, clinical, and interface implementation efforts. We followed the clinical implementation. Virtual “Clinical Information Calls” led by external facilitators and attended by internal facilitators pre-figured the in-person “Clinical Process Design Workshop (CPDW).” The Clinical Information Calls continued through an intensive 2-h Skype “Train the Trainer” that was followed by the culminating event, the in-person inauguration of Tele-CC services, or the “Go-Live.”

The Tele-CC nurses had all worked as bedside ICU nurses. They understood the protectiveness and emotional attachment characteristic of relationships between nurses, patients, and families in ICUs; they also understood that offering critical care virtually could disrupt relationships at the bedside. This manuscript will trace how Tele-CC and ICU staff negotiated mundane connections occurring within the daily flow of Tele-CC and ICU staff in and out of patients’ rooms. In the STS case study presented in this manuscript, we will model how to use STS and pay attention to aspects of material culture that may help implementors better understand and intervene upon Tele-CC implementation barriers.

## Methods

### Overall aim & Design

Elements of our ethnographic process evaluation [9] have been laid out in a previous manuscript [46]; the supporting research was approved by the University of Iowa Institutional Review Board (IRB # 201311734). The clinical leader of the implementation (RP) formally introduced the evaluation team (HSR, JM, JVT, JF) at the Clinical Process Design Workshop, which served as a kick-off meeting for each new round of sites. During subsequent site visits and in conversation with participants, the evaluation team introduced themselves as social scientists. We indicated that we would report our findings to the VA Office of Rural Health, which was funding the evaluation of the implementation of Tele-CC in rural sites across the United States (Award # 14385).

Over the course of 16 months, the evaluation team conducted participant observation, including producing fieldnotes [47], document review, and interviewing using qualitative techniques (e.g., root questions) [48]. We analyzed our data by first organizing segments of fieldnotes and interview transcripts according to categories [49] of implementation strategies and then according to complementarity of information across types of data (observations and fieldnotes, documents, and interviews) collected longitudinally [4], in order to build a case study in the tradition of STS. Across our data collection and analysis, we used verification strategies [50] in order to ensure the reliability and validity of our process and findings.

In this article, we will trace how external facilitators used planning and educating implementation strategies (e.g., building buy-in, developing relationships, developing materials, and educating) to normalize Tele-CC. Specifically, we will focus on the conversations around the doorbell (a chime that would ring over the speaker in the patient’s room), a feature of the Tele-CC that Tele-CC staff use to mark their impending presence in the ICU room. The focus on the material culture of the doorbell developed during the iterative analysis process (see analysis section below). We used ethnographic data collection techniques through time, as well as across sites at one point in time. As a result, we were able to produce stratigraphic observations and horizontal exposures of the tensions around the doorbell, and thus generate a partial ethnography of the uneven normalization of Tele-CC in VA.

### Setting & characteristics of participants

Our continuous virtual ethnographic engagement with the implementation of Tele-CC was punctuated by in-person site visits and presence at training events. The evaluation team was included on the list of attendees at virtual events and meetings, alongside internal and external facilitators. Prior to site visits, internal facilitators and ICU staff were approached via email regarding interviews with the evaluation team. A convenience sample of external and internal facilitators, as well as ICU staff, was selected based on their presence and involvement in the implementation of Tele-CC. Participation in interviews with the evaluation team was not mandatory; however, no one outright refused to participate. External and internal facilitators from the Tele-CC and ICUs included intensivists, advanced practice nurses, and nurse managers. ICU staff included intensivists, hospitalists, nurse managers, nurses, telemetry techs, and nursing assistants across all shifts. This article reports on fieldnotes from virtual events, including the Clinical Implementation Calls and Train the Trainer event, as well as our fieldnotes and interviews at in-person events, including the Clinical Process Design Workshops (CPDW) and sites visits at three ICUs that adopted Tele-CC.

### Data collection

Three ethnographers, with post-graduate degrees in geography, public health, and anthropology (JM, JF, and JVT, respectively) led the data collection efforts. We collected fieldnotes throughout the implementation process. During the virtual events (Clinical Information Calls, Train the Trainer), we called into the meetings and were largely silent; our presence was registered on the attendee list. At in-person events (CPDW, Go-Live), we embedded ourselves within small groups and participated with them in whatever activities were taking place. At 6-months post-implementation, we returned to the sites and conducted semi-structured interviews with ICU staff and internal facilitators.

### Observations and Fieldnotes

During virtual events, JF and JVT observed conversations between external facilitators and internal facilitators. Conversations revolved around technical readiness, information about dates and times of upcoming events (CPDW, TTT, Go-Live), questions from the internal facilitators, and, post-CPDW, an in-depth review of each workflow layering Tele-CC into ICU practice. During the CPDW, we took notes on the lecture accompanying the PowerPoint Presentation, questions posed by internal facilitators, conversations among internal facilitators, the simulation demonstrating how the Tele-CC can assist ICUs, and the process of developing workflows. During Go-Live events, we took notes on small-group training sessions and simulations. In total, we conducted 101 h of observation (42 h during the Clinical Information Calls, 4 h during the Train the Trainer sessions, 35 h at the CPDWs, and 20 h at the Go-Live events).

### Document retrieval

JF and JVT collected copies of distributed materials, including PowerPoint presentations, workflow diagrams, training templates, brochures for doctor orientation and patient and family guides, as well as copies of the scripts for training simulations. In this article, we focus specifically on the elements of the documents that focused on the doorbell, including several PowerPoint slides, and the workflow diagrams around “Camera Etiquette” (see Additional file 1).

### Semi-structured interviews

During Go-Live, and then at 6-months post implementation, JM and JVT conducted semi-structured qualitative interviews using qualitative techniques, including linguistic intentionality, root questions, and grounded probes, in order to solicit multiple perspectives and make space to question assumptions [48] (Additional file 2). To promote conversation and reflexivity [51], two researchers co-led each interview. At the initiation of Tele-CC services at the site, we asked questions about the structure and function of the ICU and the patient population, preparations they had made for the implementation of the Tele-CC, as well as their knowledge about the Tele-CC. At 6-months post-implementation, we asked questions about staff expectations and perceptions of the Tele-CC, as well as how they had used it. Interview duration was based on participant availability; however, no interview lasted longer than 60 min. Interviews were audio recorded, transcribed by trained transcriptionists, and uploaded into MAXQDA for analysis [52]. Transcripts were not returned to participants for comment or correction, however we did do some member-checking [53] during repeat interviews either with the same individual, or individuals who occupied the same role, as we visited the same three ICUs at Go-Live and then 6 months post-implementation. Details about these interviews are reported in an earlier manuscript [46]; additional information is included in Table 1 (below).

**Table 1:** Implementation Landscape

Normalization Work	Implementation Process	Number of Participants				Implementation Role	Location	Ethnographic Presence
		Hub	Site 1	Site 2	Site 3			
			Jan-Jun 2017	Jan-Jun 2017	Jul-Nov 2017			
Planning, Enrolment	Clinical Information Calls		1-5/ call	1-5/ call	1-5/ call	External Facilitators	Virtual	Observation Fieldnotes Document retrieval
			2-4/ call	2-4/ call	2-4/ call	Internal Facilitators		
			0	0	0	ICU Staff		
			Feb 2017	Feb 2017	Sept 2017			
Planning, Initiation	Clinical Process Design Workshop (CPDW)		3	3	4	External Facilitators	In-person at the Tele-CC Hub	Participant Observation Fieldnotes
			6	6	6	Internal Facilitators		
			0	0	0	ICU Staff		
			April 2017	June 2017	Nov 2017			
Education, Legitimation	Train the Trainer		2	2	3	External Facilitators	Virtual	Observation Fieldnotes Document retrieval
			8	8	n/a	Internal Facilitators		
			0	0	0	ICU Staff		
			Jun 2017	Aug 2017	Nov 2017			
Education, Activation	Go-Live		5	5	4	External Facilitators	In-person at the ICU site	Participant Observation Interviews
			2	4	3	Internal Facilitators		
			27	14	15	ICU Staff		
		Sept 2017	Dec 2017	Jan 2018	Jun 2018			
	6-months post implementation site visit	16				Tele-CC Staff	In-person at the ICU site and Hub	Interviews
			2	2	2	Internal facilitators		
			14	13	18	ICU Staff		

### Data analysis

The analysis described here was conducted for the specific objectives noted above and reflects a small part of the larger evaluation of Tele-CC implementation in VA conducted by our team [46, 54, 55]. Throughout our evaluation, JM, JF, and JVT used qualitative data verification strategies, to ensure the reliability and validity of our data collection and analysis process [50]. We have also been guided by Normalization Process Theory [34–36]; for this analysis JVT, JM, and JF categorized each implementation process by the normalization work involved: enrolment, initiation, legitimation, or activation. These details are laid out in Table 1.

After organizing the data in this way, JVT deductively coded [49] fieldnotes according to the implementation strategies of planning and education (i.e., building buy-in, developing relationships, developing materials, and educating) [45]. While deductively coding, JVT found that one of the most intact examples of a workflow, the one for “Camera Etiquette,” was also an element of the implementation for which we had a diverse pool of data (fieldnotes, interviews, and documents). JVT conducted lexical searches across fieldnotes and interviews for “workflow” and “camera.” JVT organized the coded segments that included the terms “workflow” and “camera” chronologically, according to elements of commitment work, and noticed a particularly potent interaction between an external facilitator and an internal facilitator around the idea of the doorbell. To draw out the potential tension, and collect data from as many voices as possible, JVT conducted another lexical search for “doorbell” in interviews with all staff interviewed 6-months post-implementation at the sites. Throughout this analytic process, JVT was in conversation with JM about the application of Normalization Process Theory as an etic frame, as well the possibilities afforded by approaching the data from the perspective of science and technology studies (STS). As a result, JM and JVT wrote the article in an iterative process, in conversations shaped by effective qualitative interview techniques designed to encourage reflexivity [51] and thus draw out the richness of the connections highlighted by the different forms of data (fieldnotes, documents, interviews) collected over time [4]. We refined the discussion and conclusions through discussions and writing with the clinical leader of the implementation (who was also the Medical Director of the Tele-CC) (RP), the external educator who co-led the Go-Live trainings (who was also an APRN in the Tele-CC) (LF), and a subject matter expert who was a former ICU nurse and current VA Rural Health Scholar (JW).

## Results

Following the doorbell through the layers of the implementation process, and then across three sites at 6-months post-implementation, we exposed how different and divergent notions of surveillance grew up through the implementation of Tele-CC. We pieced together this narrative about surveillance based on our ethnographic method of data collection. Concerns about surveillance are a barrier to staff acceptance of Tele-CC, and to understand how surveillance is a barrier, we can map the materials through which surveillance comes to matter. To tell stories about surveillance, ICU and Tele-CC staff implicated brochures, cameras, buttons, chimes, motors, baths, curtains, courtesy, nighttime, spying, post-operative confusion, and voices.

Tele-CC staff used the doorbell to signal their entrance into the patient’s room. Following the chime, the camera would turn on and swivel around to face the patient’s bed and the face of the Tele-CC clinician would appear on the computer monitor. In contrast, ICU staff used a combination of slower, protracted signals, including knocking on the door, or tentatively moving the curtain, in combination with verbal cues to enter a patient’s room. The chime of the doorbell and the inevitable whir of the camera’s motor as it rotated toward the patient were new sounds for ICU staff. In talking about these sounds, ICU staff found a way to express their concerns about surveillance and privacy, for their patients, for their relationship with their patients, and for themselves.

### Stratigraphic (longitudinal) observations (site 3 through the implementation process)

During Clinical Information Calls, in working through the “Camera Etiquette” workflow, internal facilitators and external facilitators spent time addressing questions about standardizing times when Tele-CC staff planned to round on ICU patients, obtaining verbal agreement from the patient for the Tele-CC to camera in to their room, potential equipment malfunctions and, specifically, the doorbell. Over the course of several calls, the external facilitators and internal facilitators worked to refine the workflows to best reflect how the Tele-CC could be “layered in” to the existing practices of the ICU. During the Clinical Implementation Call on July 11, 2017, during the discussion of the workflow entitled, “Camera Etiquette,” Patricia, one of the internal facilitators from Site 3 queried Morris, one of the external facilitators about the doorbell. The exchange is transcribed from fieldnotes below:

*Patricia (Site 3): Is there a bell you ring prior in case the patient is being bathed?*

*Morris: Yes. You’ll hear the motor of the camera move. We’ll click and show our picture. Somewhere in there, they will press a button and it will ring a doorbell.*

*Patricia: Perfect*

*Morris: At night, we don’t do that. We surveyed our customer clinicians.*

*Patricia: Did you have to put up a disclaimer or any notification that cameras are being used?*

*Morris: We give a brochure to the staff. It is a VA Telehealth rule that all patients have to consent to the video. Our nurses have a script of what they say and they’ll get consent for the audio portion of the ICU. Less than 1% of all patients refuse the [Tele-CC]. No reason to refuse, they are getting additional physicians looking over them. Does not preclude your nurses from connecting with us, just we can’t camera into the room.*

(Fieldnote, Clinical Implementation Call, July 11, 2017; all names are pseudonyms)

The import of Patricia’s question, “*Is there a bell you ring prior in case the patient is being bathed*,” and Morris’s response, “*You’ll hear the motor … we’ll click and show our picture … they will press a button and it will ring a doorbell*,” is not clear until the Clinical Process Design Workshop (CPDW) event 3 months later, when we participated in a conversation with Patricia and her colleague to create workflows. Our fieldnotes read,

*after [an external facilitator] explained that the doorbell would sound after the [Tele-CC] nurse was in the process of camera-ing in, and that bedside staff wouldn’t have direct decision making about whether or not to permit this access … the major concern she [Patricia] mentioned was privacy for patients. [Her colleague from Site 3] replied that it would probably be similar to how people walk in and out of rooms at the hospital when rounding on patients, potentially walking in on them in moments when privacy would have been preferred. Patricia responded to this by saying in a flat tone, “Not in my ICU.”* (Fieldnote CPDW, September 2017)

Similarly, the significance of Morris’s clarification that “*at night, we don’t [ring the doorbell*],” was not obvious until the Go-Live event at Site 3 (4 months after the CPDW). In an interview, Patricia spoke with us about how,

“they [the Tele-CC staff] don’t like to ring the doorbell, middle of the night to check on the patient. I want them to and they went back and forth about this … it’s like I kept saying to them, when I go into a patient’s room, I knock on the door. So that’s why I want you to ring the doorbell … you know, if I’m going into a patient’s room just with the curtains drawn, I’m gonna knock, I’m gonna say, ‘This is the nurse … [okay] if I stick my head in?’ You know? And they’ll say yes or no … but that’s the same thing I want the courtesy of the, of the doorbell.” (Site 3 T1, RN ICU)

During Go-Live, Morris oriented staff to Tele-CC through training sessions with small groups. After a brief lecture about the history of Tele-CC, Morris encouraged bedside staff to practice engaging with the Tele-CC by hitting the green button newly installed in each ICU room. In encouraging engagement with the Tele-CC, Morris specifically mentioned the doorbell. A fieldnote from one of these small groups describes his characterization of the doorbell:

*Morris explains that … the hub staff can call in to the room from their end but will not do so without using a “doorbell” to buzz in to let staff and patients know that they are doing so. The camera will also rotate into the room to alert patients and on-site staff when hub staff call in. Morris has both [trainees] practice answering potential questions from patients and visitors about the cameras and the Tele-CC program along the lines of: “What is that thing? Why is it in here?” Morris also asks them to respond to a patient saying, “I don’t want it spying on me,” to which [the trainees] reply that it won’t do that.* (Site 1 T1, Fieldnote)

Morris’ admonition to the trainees presages the implication of Patricia’s question about “*putting up a disclaimer or any notification about cameras,”* which became visible 6 months post implementation (June 2018). Patricia had left her position, but another internal facilitator from Site 3, Forrest, who had attended the Clinical Process Design Workshop with Patricia, relayed how,

“[if] there’s no nurse in the room and there’s the [Tele-CC] nurse practitioner, you know, and the patient’s like, ‘What? I can’t hear you,’ … [and] we [the ICU nurses] didn’t hear the doorbell and then we didn’t answer it … I think that those are the kinds of opportunities we have to ensure that it’s a good patient experience … Many of our patients come post-operatively where they’re not able to be oriented [to the Tele-CC] and they could be very confused … that all of a sudden somewhere out of space a voice is coming from this thing on the wall” (Site 3 T2, MD ICU)

Retrospectively piecing together the arc of the implementation process by threading a narrative through mentions of a material object (e.g., a doorbell) was a way to re-situate ourselves in the flow of the original timeline of implementation. We developed a sense of what the doorbell was connected to (i.e., concerns about surveillance). As a result, we anticipated that looking for when people talked about the doorbell during our interviews 6-months post implementation might help us understand how conversations about surveillance changed, and also how these conversations differed across sites. Our “good case history” helped us contextualize and better understand discussions at 6-months. Looking retrospectively was a way to understand prospectively.

### Horizontal (Cross-Sectional) Exposure (6-months post implementation at Site 1, Site 2, and Site 3)

Each of these threads of Patricia’s concerns were borne out amongst the ICU staff at six-months post implementation with bedside staff at Site 3. Nurses at Site 3 relayed how,

“They’re supposed to ring the doorbell. I don’t know if we don’t hear the doorbell? But we certainly don’t know when they’re gonna just pop in, usually. (Site 3 T2, RN2)

“We were under the impression … when it first got initiated, there was going to be a doorbell before any camera turning, any monitor pop … and they were supposed to talk, for instance, “Is it okay if we come in?” and that is not the case.” (Site 3 T2 RN5)

“There’s been at least three instances where they have just come in while I’ve had a patient either on the commode or standing there urinating, and I was under the impression that we could deny them entry—[P2: (overlapping) That they’re supposed to … ring a doorbell.] … Well, the doorbell rings, but then it just turns off. [P2: Oh, I don’t even hear it, yeah] … Y-you got the green button, but there should also be a red button, so if you hear the chime, you can push the red button and they WON’T come in.” (Site 3 T2 RN6 & RN 7)

Not all ICU nurses shared the perspective of the nurses at Site 3. At Site 1, we engaged two bedside nurses, who had not been internal facilitators during the implementation, in the following conversation about the doorbell at 6-months post implementation:

“[I1: We’ve heard from several different folks we’ve talked to across sites that there’s anxiety about [Tele-CC] just camera-ing into the room without calling first or ringing the doorbell. Because you had that previous set of interactions with them, has that anxiety waned?] P1: It does still surprise us sometimes when we hear a voice in there and we’ll think, “Oh, I didn’t hear the doorbell,” [I1: Yeah.] you know, so [P2: (Overlapping) Hmm yeah] sometimes the doorbell … doesn’t ring … and so they’ve [P2: Yeah.] caught us off-guard. Sometimes we’ll be in there moving a patient or something and they’ll [P2: Oh!] uh (chuckles) … We know that they will um pop in between, say, eight o’clock and nine or ten [P2: Mm-hmm.] and do an assessment on the patient, so when we hear that we’re used to hearing ‘em, but we just don’t, a lotta times don’t hear the doorbell

[I1: I see so when you hear ‘em, what do you hear?] P1: Just voices talking … They talk to the patients … [and we wonder to each other] Is that your patient? Who are they talking to? (chuckles) And then we realize it’s probably [Tele-CC] that they’re talking to

[I1: Okay so walk me through that.] P1: (Laughs) Well just sometimes it, you know, it’s eight, nine o’clock and you’ll hear someone that you-- and you’re wonderin’, is their family member in with that patient or, you know, something like that and then we kinda listen to the conversation a little bit because the [Tele-CC] has a sound, you know, [P2: Hmm.] it’s uh-- doesn’t it? Doesn’t it? It’s different than just some-- just us— [ P2: (Overlapping) Yeah, tell it’s on a speaker.] P1: Yes … Kind of an echo. [P2: Like, now if you’re listening to a radio or something, you can tell they’re-- --not right beside you. It’s--] P1: It’s a different kind of sound [P2: Mm-hmm.]. P1: It’s a different conversation than us just talking... we don’t hear it all the time, you know, and so we-we haven’t learned to assimilate it into our-our book of sounds

[I1: What does that feel like to know that there’s another presence kind of like paying attention to all of the … ] P1: (Pause) At first, it was a little uh anxious, or a little irritating just because someone else is coming in and havin’ eyes on your patient, but their-- they don’t, they don’t butt in [I1: Okay.] is what I have found. They don’t butt into the care that I’m giving.” (Site 1 T2 RN Night Shift)

At Site 2, nurses we spoke with did not mention the doorbell when they reflected on how Tele-CC staff entered patient rooms and initiated conversations. One nurse remembered how,

“I mean uh you know [they have] popped in and you know ‘how’s he doing and how’s this and how’s that.’ And converse with the people who are there. I mean I, like I said I’m fine with it. Some people I think, were very apprehensive about it. But even the people that were very apprehensive, I think that after they got used to it, they didn’t care. I mean [the Tele-CC staff] would go on ahead and they were popping in on the patients. And you know when someone’s got their door closed like over here, and the family member’s in there and that shade is pulled. Guess what? You know [Tele-CC] pops in and of course they’re gonna flag us if there’s a problem. So that’s a good thing to have.” (Site 2 T2 RN3)

Ultimately, staff at Site 3 wanted to be able to limit Tele-CC virtual entry into their ICU rooms. Staff at Site 1 and Site 2, despite having some similar misgivings about the shifting dynamic of relationships between the Tele-CC, ICU, and patient, did not feel the same way. At Site 3, the conversation hardened around hearing or not hearing the doorbell, and wanting the opportunity to hear the doorbell. At Site 1, the staff also missed the sound of the doorbell, but focused instead on how the “different kind of sound” produced by the Tele-CC signaled “a different conversation” at the bedside. Staff at Site 2 did not mention the doorbell when they recollected interactions with the Tele-CC, but they also noticed the sound of the conversation between the Tele-CC and patient; what is more, they perceived how the Tele-CC could help them circumvent barriers to entering the room (e.g., closed doors, pulled shades) that the patient and family sometimes imposed.

## Discussion

The ICU is a place full to bursting with sounds. Patients risk developing “ICU delirium” as a result, in part, of the sounds associated with continuous monitoring of vital signs [56] and some nurses we spoke to talked about having a “book of sounds.” We witnessed nurses respond strategically to different sounds; turning off some “alarms,” but noticing immediately and acting decisively when a sound indicated a patient was in trouble. The sound of the doorbell was new. As a noise in the ICU, the chime was an unfamiliar aural presence [57, 58] that inadvertently encouraged nurses to notice other foreign presences accompanying the implementation of the Tele-CC.

By “recovering [the doorbell] archaeologically and interrogating [the doorbell] ethnographically” [21], we have demonstrated the utility of the STS case study as a contribution of ethnography to implementation science. While ethnography exposes the mundane particularities of an implementation, science and technology studies (STS) helps us think about how those things come to matter. Specifically, STS case-studies contribute to the literature on longitudinal qualitive research (LQR) in implementation science, including pen portraits [4] and periodic reflections [3]. Like periodic reflections and pen portraits, the STS case-study provides a way to engage with the complexity of an implementation process by tracing changes over time through interviews and observations. However, the form of an STS case-study is unique. Rather than a clean case summary, it is more like a complex case history full of the mundane bits and pieces like those pointed out by Mol and Law; here, rather than “beans, blood, [and] table companions,” we followed brochures, cameras, buttons, chimes, motors, curtains, and voices [16].

Both ICU and Tele-CC staff enter patient rooms, but they do with different tools, with different “stuff.” Bedside nurses have a curtain or a door; Tele-CC nurses have a camera that turns around and a chime they call a “doorbell.” Entering patients’ rooms implicates cameras, chimes, motors, curtains, and voices, and negotiations about how to use this stuff, sparks concerns about how ICU and Tele-CC nurses differently acknowledge movement from the communal space in the ICU to the intimate space of the patient’s room. The material stuff associated with the presence of the Tele-CC (e.g., the camera, speaker, and monitor) are already located in the patient’s room, and so we must think differently about how a Tele-CC nurse could be noticed moving from communal to private.

Though labor intensive, the components of ethnography (e.g., participant observation, fieldnotes, archival research, and interviews) generate a field of data that can be analyzed archaeologically (e.g., across and within sites, at one moment in time and over time) and as a consequence allow us to notice tacit and implied beliefs that impact an implementation process. As researchers, we did not initially know to ask about the doorbell, and it was only after combing through our fieldnotes and collected documents that we were able to trace conversations about the doorbell to planning and educating materials pre-implementation, and then forward to conversations among ICU staff 6-months post-implementation. Anchored by the material, the heterogeneity of an STS case-study generates questions (e.g., why did Patricia demand the doorbell be rung at night? Is she concerned about privacy for her staff, or the patients, or both?) and encourages exploring differences (e.g., how did nurses at Site 1 let go of wanting the sound of the doorbell and embrace the different sounds of the Tele-CC? When did the nurses at Site 2 begin to see the Tele-CC as a way for them to see into the room?). Begun early enough, the STS case-study method, like periodic reflections, can serve to iteratively inform data collection for researchers and implementors.

Tele-CC staff need a metaphor that positions the Tele-CC differently vis à vis the ICU (e.g., not a doorbell, but maybe an “arrival chime”). Terming the sound a “doorbell” implies that ICU staff may not permit Tele-CC to enter the room, much like when someone rings a doorbell at a house and the owner chooses whether to invite entry. In our context, the Tele-CC are part of the standard of care (i.e., Tele-CC cannot be denied entry into a patient’s room). Tele-CC staff recognize that ICU staff have a strong sense of autonomy in their practice and they wonder if using the term “doorbell,” and thus (incorrectly) implying that ICU staff can deny Tele-CC staff entry in to the room, creates uncertainty among ICU staff related to their own autonomy and the authority of the Tele-CC. The goal is to initiate contact with a sound that signals collaboration and partnership. Future research should explore how one negotiates virtual entry to an intimate, private space in a way that fosters teamwork.

### Limitations

Our study has several limitations. First, teamwork between ICU and Tele-CC staff is so complex that 6-months is not enough time for Tele-CC and bedside staff to become familiar or comfortable with each other; in fact, it could take longer than 6 years to build trustful relationships [59]. Our data collection plan ended at 6-months post-implementation, so we did not have the opportunity to observe and learn about how staff interacted with the doorbell in the context of more trusting relationships between the ICU and Tele-CC staff. Secondly, we have no information about how patients perceive the sound of the doorbell. Finally, we do not have data gleaned from interview guides informed directly by our new understanding of the import of the doorbell. If we had the opportunity to go back to these sites, we could ask them questions that might draw out this information. However, using the STS-case study method, we were able to denote a pattern that may indicate that staff who are normalizing the sounds associated with Tele-CC may be exhibiting higher levels of acceptance of Tele-CC a part of their practice.

## Conclusions

The STS case-study is a tool for implementors to use when a piece of material culture is an essential component of implementation. In the context of an ethnographic process evaluation of the implementation of Tele-CC services in Department of Veterans Affairs Medical Centers, the STS case-study helped us realize that we must think differently about how a Tele-CC nurse could be noticed moving from public to private space. The next step in the development of the STS case-study research method is to develop tools that will guide implementers through the STS case-study method to determine systematically how material culture can reveal implementation barriers and direct attention to potential solutions that address tacit, deeply rooted challenges to innovations in practice and technology.

## Supplementary Information

**Additional file 1:.** Workflow for “Camera Etiquette”

**Additional file 2:.** Interview Guides

## Data Availability

The datasets used and/or analyzed during the current study are available from the corresponding author on reasonable request.

## Abbreviations

CPDW: Clinical Process Design Workshop
ICU: Intensive Care Unit
LQR: Longitudinal Qualitative Research
STS: Science and Technology Studies
Tele-CC: Tele-Intensive Care Unit (previously abbreviated as Tele-ICU)
TTT: Train the Trainer
VA: Veterans Affairs
