# Supplementary figures and images for: The STS case study: an analysis method for longitudinal qualitative research for implementation science
Source: BMC Med Res Methodol. 2021 Feb 5;21:27. doi: 10.1186/s12874-021-01215-y (PMC7866713; doi:10.1186/s12874-021-01215-y)

*Additional file 1: Workflow for “Camera Etiquette”*

March 14, 2017


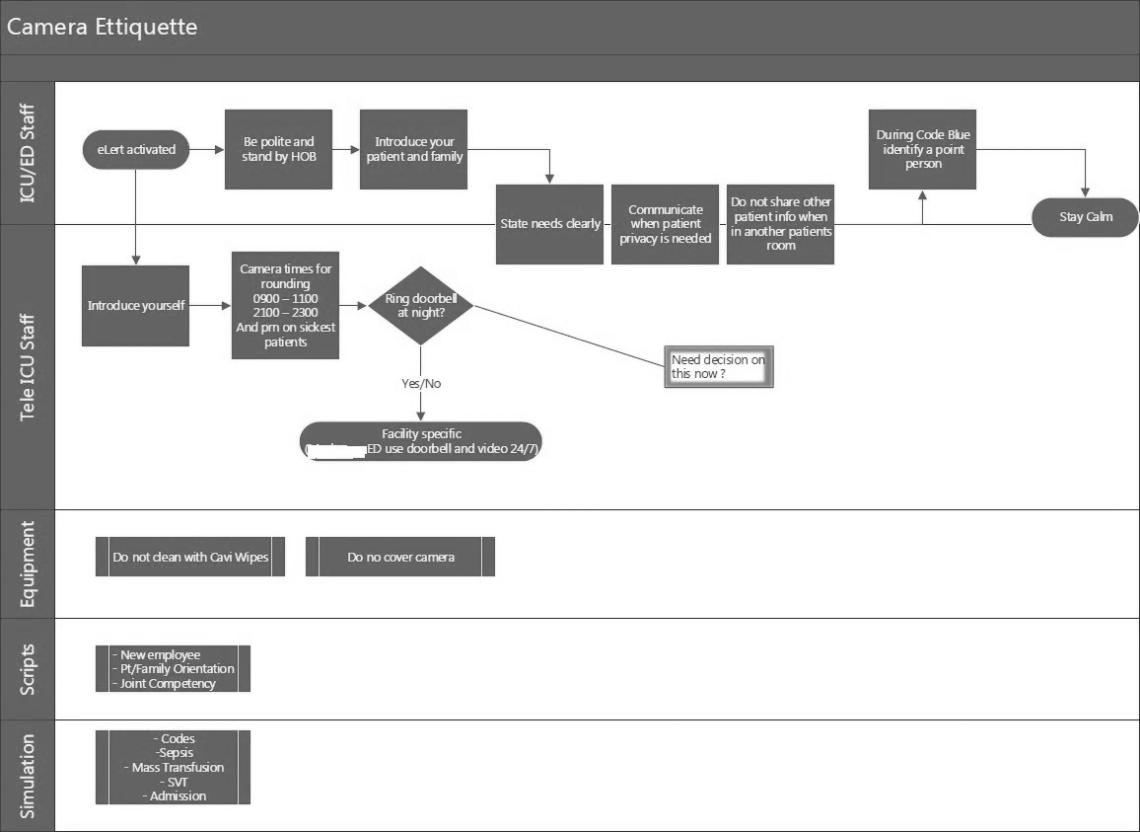


March 28, 2017


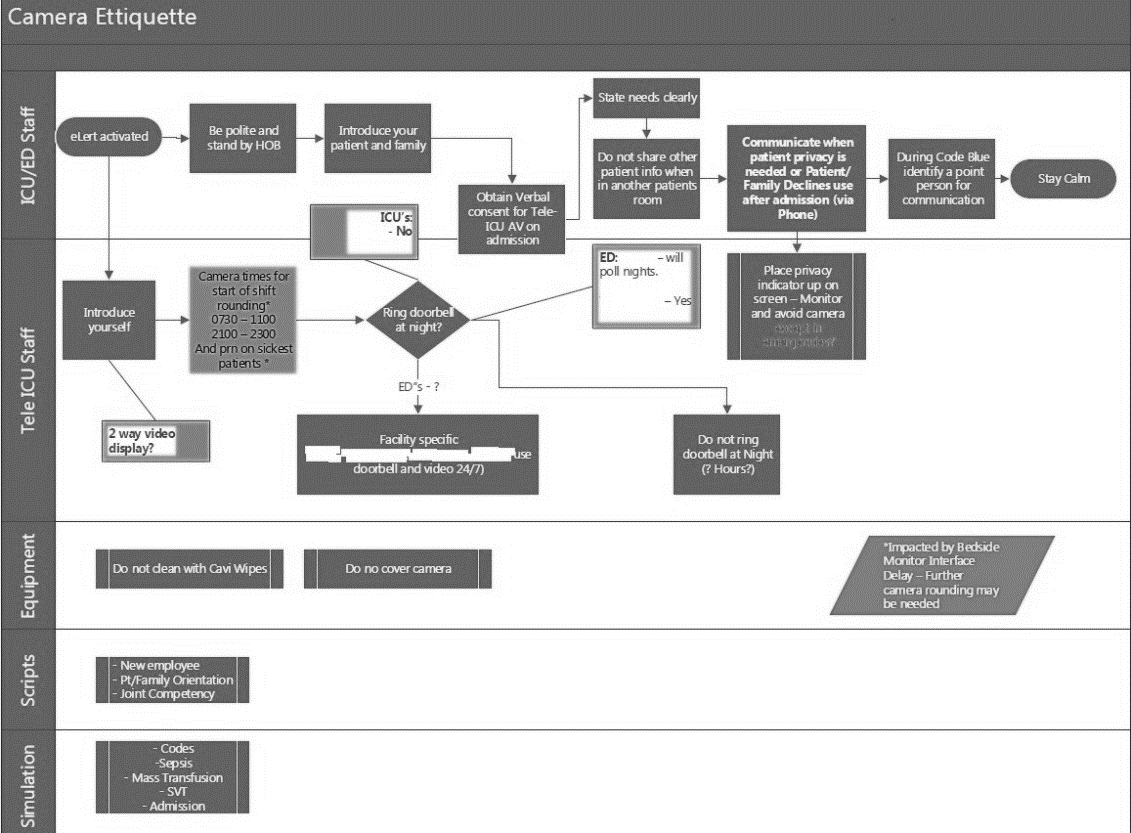

Supplement: Supplementary file 1 — Additional file 1:. Workflow for “Camera Etiquette” [file 12874_2021_1215_MOESM1_ESM.docx]
