# Supplementary material for: The STS case study: an analysis method for longitudinal qualitative research for implementation science
Source: BMC Med Res Methodol. 2021 Feb 5;21:27. doi: 10.1186/s12874-021-01215-y (PMC7866713; doi:10.1186/s12874-021-01215-y)
Supplement: Supplementary file 2 — Additional file 2:. Interview Guides [file 12874_2021_1215_MOESM2_ESM.docx]

*Additional file 2: Interview Guides*

*Semi-Structured Interview Guide for Site Visits at Go-Live*

1. Tell me about your day. What goes on in your mind?
2. How would you describe the flow of work in your unit? Who does what kind of work in your unit?
3. Where do patients who are admitted to your ICU/ED come from? Where do they go when their time in your unit is over? Are they typically transferred or discharged? To where and why?
4. There is a lot of technology in the ICU/ED setting. How do you use it to understand what’s happening with the patient? Describe how your facility and your unit is preparing for Tele-ICU.
5. Describe the level of familiarity you have with Tele-ICU.
6. Whose needs are met by Tele-ICU, in your opinion? Who benefits from its presence?
7. Describe how Tele-ICU might impact the flow of your day and the flow of your unit. How do you think you might use it?
8. Why do you think the VA is investing in this system? Why do you think your facility is investing in Tele-ICU?

*Semi-Structured Interview Guide for Site Visits 6-months post-implementation*

**PART 1: BROAD QUESTIONS**

*We’d like to start with some broad questions about Tele-ICU.*

*We are interested in your expectations and your perceptions.*

*We’d also like to know how you’ve been using Tele-ICU.*

- **Expectations and Perceptions**
  - Overall, how satisfied are you with the Tele-ICU. Tell me more about that
  - How do you describe the Tele-ICU? What does it mean to you or your practice?
  - What has surprised you about Tele-ICU? Examples
  - Why did your unit want Tele-ICU support? Has your facility identified specific problems that Tele-ICU is intended to solve?
  - When we were here, you mentioned that a main motivation for bringing in Tele-ICU was XXX. Has Tele-ICU benefited your ICU specifically in this way? Example
    - - XXX = backup/safety net/second set of eyes, continuity b/w shifts, and coverage
    - In what ways have your expectations been met? Have not been met? Examples
- **Utilization**
- Have you used Tele-ICU? If yes -
  - When an interaction with the Tele-ICU has gone well, what happened? Provide an example.
  - When an interaction with the Tele-ICU has gone less well, what happened? Provide an example.
- Let’s talk about interactions you’ve had with the Tele-ICU. What events have triggered the most calls from the hub to your unit?
- What events have triggered the most calls from your unit to the hub?
- How has advice from Tele-ICU staff been received in the ICU?
- How do these types of reactions compare to advice you’d receive by a nurse or physician at the bedside? Or a consult over the phone?

**PART 2: QUESTIONS ALIGNED WITH THE SURVEY (for Site C Time 2)**

*We sent you a survey 4 months after Tele-ICU was implemented and asked you a range of questions.*

*We’d like to check in with you about some of those questions and generally just follow up on our earlier visit and see how things are going.*

*We hope that you can help us understand some of the longitudinal relationships that we are tracking.*

- **Continuity (ambivalent), Quality (leans positive), and Safety of patient care (ambivalent/leans positive)**
  - Has Tele-ICU changed how you think about patient care practices? About your profession?
- What impact, if any, do you think Tele-ICU will have on patient care and patient outcomes? How your unit functions and performs? E.g., Standardization of care? Alerting staff to patient problems? Providing diagnostic and treatment consultation? Improving patient outcomes?
- **Interrupts daily workflow (ambivalent)**
  - Help me understand how this technology might or might not interrupt your daily workflow.
    - With access to Tele-ICU, has your daily practice changed? If so, how? If not, why not?
    - Have you experienced more or less interruptions in your daily practice?
- **Improve communication in caring for a patient (ambivalent/leans positive)**
  - How is the Tele-ICU staff like a member of the care team? And how are they not? Examples
  - What is communication like between bedside/Tele-ICU nurses and between nurses and physicians (and other roles)? Are there processes in place to structure your communication or improve information flows? (communication and information flows)
    - How about the coordination of care? How is information shared? (coordination)
    - How are decisions made? Who is included in making decisions or providing input? (decision making)
    - When a problem arises, how do you typically address it? (problem solving)
  - When we were here, it was suggested that there were some local team issues among providers. How has Tele-ICU impacted these issues?
    - Teamwork among nurses was identified as one of the strengths of your unit. In what ways has Tele-ICU impacted nurses’ teamwork?
- **Helps with feeling less overwhelmed (ambivalent)**
  - Has Tele-ICU made your job easier or harder? Why?
  - How do you expect your ICU to adapt to Tele-ICU support over time?
    - What organizational changes have occurred as the Tele-ICU-ICU/ED? (e.g., shifts in hiring practices, shifts in policies)
- **Will save time (ambivalent)**
  - Can you provide some examples of how you have used Tele-ICU to perform different aspects of your job? (e.g., how frequently you draw labs, chart, perform patient personal care and pressure ulcer prevention (e.g., repositioning patients), but also “off camera” activities like hand washing and waste and drug disposal)
  - In what ways has Tele-ICU affected your workload?
    - Has Tele-ICU affected the time you devote to tasks?
- **Improve patient satisfaction (ambivalent) & Improve patient’s family’s satisfaction (leans positive)**
  - Whose needs are met by Tele-ICU, in your opinion? Who benefits from its presence, and how?
    - How well do you think Tele-ICU meets the needs of the individuals served by your organization? [CFIR]
    - How have patients responded to the remote ICU system? (e.g., Transmission of their medical information, the ability of remote care providers to video and zoom in on their body)
    - How have families responded?

Quant (patients) vs Qual (providers) / staff vs patients / noticed consistency in pre-/post-

- **Decreases patient privacy (ambivalent)**
  - Help me understand how this technology might or might not impact patient privacy.
- **Worry about feeling spied upon (leans negative/ambivalent)**
  - Are there potential issues related to your work responsibilities or to the technology that you are concerned about?
- **Educational resource (leans positive)**
  - Help me understand how this technology might or might not work as an educational resource.

**PART 3:** *(FOR IMPLEMENTATION TEAM, MANAGEMENT, LEADERSHIP)* **QUESTIONS ABOUT THE IMPLEMENTATION PROCESS**

*We’d like to end with some questions about the implementation process.*

*We are interested in your expectations and your perceptions.*

- **Expectations and Perceptions**
  - Were you part of the team implementing Tele-ICU? If yes: Now that the system is live, I’d like to debrief with you about the implementation process.
    - Tell me about implementing Tele-ICU here – what challenges did you encounter, what helped facilitate implementation, in your opinion?
    - What did you do locally? How much of that was driven by Cincinnati, and how much of it was driven by you all?
    - What did Cincinnati do? Was that perceived as in or out of sync with your local context? In what ways?
    - How was Tele-ICU talked about by different stakeholders, management in particular?
  - Let’s talk about the weeks surrounding Tele-ICU’s go-live.
    - Can you describe the go-live training? The go-live event? What additional training did you receive?
    - Can you compare this to implementing other systems – is this similar or different from your typical experience, and in what ways?
    - What do you think was the impact, if any, of the trainings and go-live events, etc., on your perception or use of Tele-ICU once it went live?
- **After Go-Live**
  - After the system went live, what happened?
  - Is your unit measuring the impact of Tele-ICU, or process outcomes, such as system use?
    - What kind of information have you collected as you rolled out Tele-ICU? [CFIR] How has this information change as time passed?
    - To what extent has your organization/unit set goals for (implementing) Tele-ICU? [CFIR]
    - Have you received feedback reports about the implementation or Tele-ICU itself? [CFIR]
    - How has Tele-ICU factored into other measures that are important for your unit and/or facility? (SALE, InterQual, etc.)

**PART 4: WRAP UP QUESTIONS**

- Why do you think the VA is investing in this system?
- Why do you think your facility is investing in Tele-ICU?
- Do you think it will accomplish the goals that the VA and this facility predict that it will?

*Do you have any other comments, questions, or opinions to share about Tele-ICU?*

*I want to ask again that you keep today’s discussion confidential and I want to thank you again for your participation. Thank you.*
